# Modelling and regulating of cardio-respiratory response for the enhancement of interval training

**DOI:** 10.1186/1475-925X-13-9

**Published:** 2014-02-06

**Authors:** Azzam Haddad, Yi Zhang, Steven Su, Branko Celler, Hung Nguyen

**Affiliations:** 1 Faculty of Engineering and IT, University of Technology, Sydney (UTS), Sydney, Australia; 2 CSIRO ICT Centre, Marsfield, Australia

**Keywords:** Interval training, Oxygen uptake, Heart rate, Multi-loop integral controller

## Abstract

**Background:**

The interval training method has been a well known exercise protocol which helps strengthen and improve one’s cardiovascular fitness.

**Purpose:**

To develop an effective training protocol to improve cardiovascular fitness based on modelling and analysis of Heart Rate (HR) and Oxygen Uptake (VO_2_) dynamics.

**Methods:**

In order to model the cardiorespiratory response to the onset and offset exercises, the *(K*_*4*_*b*^*2*^*, Cosmed)* gas analyzer was used to monitor and record the heart rate and oxygen uptake for ten healthy male subjects. An interval training protocol was developed for young health users and was simulated using a proposed RC switching model which was presented to accommodate the variations of the cardiorespiratory dynamics to running exercises. A hybrid system model was presented to describe the adaptation process and a multi-loop PI control scheme was designed for the tuning of interval training regime.

**Results:**

By observing the original data for each subject, we can clearly identify that all subjects have similar HR and VO_2_ profiles. The proposed model is capable to simulate the exercise responses during onset and offset exercises; it ensures the continuity of the outputs within the interval training protocol. Under some mild assumptions, a hybrid system model can describe the adaption process and accordingly a multi-loop PI controller can be designed for the tuning of interval training protocol. The self-adaption feature of the proposed controller gives the exerciser the opportunity to reach his desired setpoints after a certain number of training sessions.

**Conclusions:**

The established interval training protocol targets a range of 70-80% of HR_max_ which is mainly a training zone for the purpose of cardiovascular system development and improvement. Furthermore, the proposed multi-loop feedback controller has the potential to tune the interval training protocol according to the feedback from an individual exerciser.

## Introduction

Heart rate (HR) and oxygen uptake (VO_2_) are key indicators of functional health status; their measurements can aid early detection of cardiac diseases [1,2]. Furthermore, these cardio respiratory endurances have long been recognized as one of the fundamental components of physical fitness.

To build effective training protocols, VO_2_ and HR are often applied to assess the exercise intensity for achieving different training goals, such as fat burning or cardiovascular system improvement [3].

One of the most effective training regimens is called interval training which was first described by Reindell and Roskamm [4] and was popularized in the 1950s by the Olympic champion, Emil Zatopek. Swedish physiologist Per OlØf Astrand provided the first scientific study [5] on interval training in 1960. Since then, it has been the basis for athletic training programs for many years.

Nowadays, interval training method becomes a well known exercise protocol which helps strengthen and improve cardiovascular fitness. Previous researchers in this area [6,7] have investigated the effectiveness of interval training and its significance in improving factors associated with O_2_ transport along with muscle uptake. Interval training has been shown to raise skeletal muscle enzyme activity [8,9], improve vascular health [10], and decrease the risk of cardiovascular-disease in obese adolescents [11]. Additionally, interval training increases proteins that transport fatty acids across the mitochondrial membrane [12]. This study aims to develop an effective interval training protocol for an individual exerciser to improve cardiovascular fitness based on modelling and analysis of HR and VO_2_ dynamics.

Interval training interleaves high intensity exercises with recovery or rest periods [13]. The idea behind this approach is breaking up a set amount of work into smaller segments rather than performing a greater volume of work at a higher intensity [5]. In addition, it is well known that exercise at varying intensities helps improve exerciser’s aerobic capacity to exercise longer [14]. A well designed interval training protocol can guide the exerciser to timely shift from low to high training intensities and vice versa within the exercise zone to achieve desired exercise targets with safe.

Any interval training protocol has at least three different periods, warm-up, exercise (switching between high intensity period and recovery period), and cool-down. The most important characters for the design of an appropriate interval training protocol are time (or distance), intensity level (speed level in our case at onset segment), time of each recovery period (offset), and number of repetitions within the exercise period. For example, both the time intervals of the onset and offset segments should be properly selected based on their transient properties to avoid reaching a steady state value during interval training.

To achieve better interval training effect, this study first investigates the dynamic responses of VO_2_ and HR by building a model, which can depict both the onset and offset dynamics. Although existing literatures [15,16] have studied the HR and/or VO_2_ responses to the onset and offset exercises, we need to re-explore the onset and offset dynamics with a certain maximum intensity (70 percent of VO_2max_) for the purpose of improving cardiovascular fitness. We noticed the distinct differences of two dynamic characteristics (time constants and steady state gains) for the onset and offset of running exercises.

A switching RC circuit model for healthy male subjects has then been developed, which can well accommodate these observed differences.

Based on this model, an interval training protocol has been proposed for this group of subjects. In order to adapt to different individual exerciser, a multi-loop integral control scheme has been proposed and achieved desired simulation results. It should be pointed out that in moderate intensity exercise the relationship between HR and VO_2_ is nearly linear [17]. As the proposed interval training protocol is particularly designed in moderate intensity range, HR can be used as an indicator of exercise strength during the proposed training.

This paper is organized as follows. Section ‘Experimental equipment and exercise protocol’ describes the experimental equipment and exercise protocol setup for model estimation. Section ‘Results and discussion’ presents the main results and discussions including the proposed switching model and interval training protocol. Section ‘Individualized adaptation for the proposed interval training protocol’ describes the adaption framework as well as the multiloop PI controller design. Section ‘Control system verification’ provides a practical method to examine and verify the functionality as well as performance of the proposed controller. Finally, Section ‘Conclusion’ gives the conclusions.

## Experimental equipment and exercise protocol

### Subjects

Eight healthy non-smoking males were invited to join the experiments. They were free from any known cardiac or metabolic disorders, hypertension, and were not under any medication. The UTS Human Research Ethics Committee (UTS HREC 2009000227) approved the study and an informed consent was obtained from all participants before each experiment. The physical characteristics of the participants are presented in Table 1.

**Table 1:** Physical characteristics

**Subject**	**Age (yr)**	**Height (cm)**	**Weight (kg)**
1	27	175	55
2	32	170	87
3	29	176	90
4	29	178	77
5	42	175	80
6	29	164	64
7	31	169	67
8	26	180	77
**Mean**	30.6	173.4	74.6
**SD**	4.7	5.0	11.1

SD: Standard Deviation.

Since both HR and VO_2_ can vary with the time of the day, emotional state, food and caffeine intake, previous activity, fatigue, temperature, humidity, altitude and dehydration [18], the participants were asked to have a light meal at least two hours before the experiment and not to engage in intense or prolonged exercise for 24 hours prior to each experiment. Environmental conditions were the same for all participants. The temperature of the laboratory was set at 25 degrees Celsius and the humidity was set at approximately 50%. All subjects were requested to be familiar with an automated treadmill before experiments.

### Experimental settings

All laboratory analyses were performed using a portable gas analyzer *(K*_*4*_*b*^*2*^*, Cosmed)*. The analyzer comes with a compatible HR probe which consists of two parts: the elastic belt containing the transmitter, and the receiver.

Using chest electrodes to monitor HR signals is considered to be valid, accurate and reliable during physical activity monitoring [2].

The K_4_b^2^ gas analyzer was used because it has previously been reported to be valid, accurate and reliable [19].

The experiments have been done on healthy however untrained subjects. A submaximal running protocol, that is practical and easy to apply, was implemented in the setup. It is required that the subjects where to perform at less than 80% of their maximum heart rate (HR_max_), this is also a recommendation for unfit people and for those with respiratory or cardiac risks [20].

The relationship between HR and VO_2_ becomes non-linear during light and very highly intense activity [2]. Therefore, and for the values of HR and VO_2_ to remain located in the linear part, a moderate speed of 9 km/h was examined and set to be the highest level in the training protocol; each participant, at varying times, reached a reading of 80% of his HR_max_ at this speed.

Each subject completed two running exercises on separate occasions. In each session and as a warming up interval, the subjects were requested to walk on a treadmill at 5 km/h for 4 minutes to avoid any kind of stiffness or fatiguing effect may occur during the running interval. Then, they were asked to run at 9 km/h for 6 minutes, before a recovery time or cooling down period of 5 minutes at 5 km/h speed.

During experiments, HR was monitored to ensure the safety of the subjects. Whenever noticeable change in heart rhythm is detected the experiment will be terminated.

## Results and discussion

### Experimental results

It is known that many internal and environmental factors may have drastic effects on the readings during the experiment [2], even when all of the laboratory conditions have been standardized and set to be the same for all participants. Some environmental and undetectable factors such as dehydration, heat loss mechanisms as well as altitude may still affect the results. To reduce the influences of these uncontrollable factors, the proposed training protocol was repeated twice and the data was interpolated, averaged and filtered.

**Figure 1:**
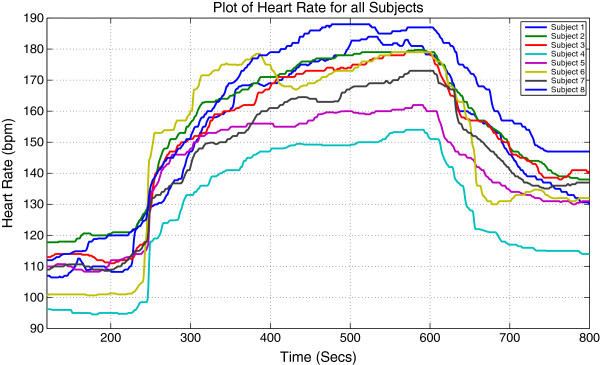
Measured HR signals for 8 male subjects running on a treadmill under a predefined protocol.

**Figure 2:**
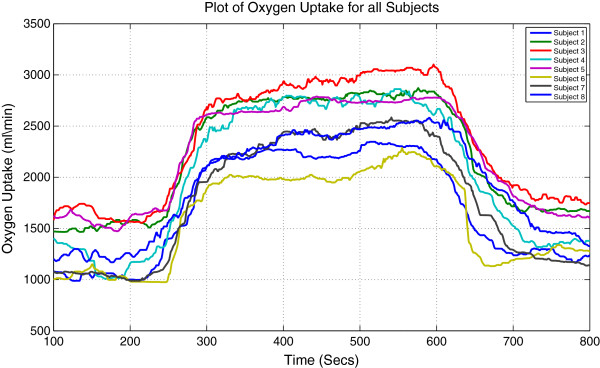
**Measured VO**_**2**_** signals for 8 male subjects running on a treadmill under a predefined protocol.**

Original signals of HR and VO_2_ for all subjects are shown in Figures 1 and 2 respectively.

According to literatures [21,22], heart rate response to exercise can be approximated as a first-order process. Since the response of VO_2_ has similar shape with HR, we model both HR and VO_2_ as first-order linear systems for both the onset and offset exercises. The first order system model can be written as follows:

(1) $$\begin{array}{l} G(S)=\frac{k}{\mathrm{\tau S}+1} \end{array}$$

Where k and *τ* are the steady state gain and the time constant respectively.

Matlab System Identification Toolbox has been used to identify the parameters of the model for each step response data. Time constants and steady state gains of HR and VO_2_ for each subject are shown in Tables 2 and 3.

**Table 2:** Estimated time constants and normalized steady state gains of HR response

**Subject**	**Onset Exercise**		**Offset Exercise**	
	**SS Gain (k**_**1**_**)**	**Time Constant (*τ***_**1**_**)(sec)**	**SS Gain (k**_**2**_**)**	**Time Constant (*τ***_**2**_**)(sec)**
1	17.2	89.4	14.6	117.9
2	14.3	62.7	12.4	117.4
3	15.7	65.6	10.4	90.3
4	13.6	52.9	9.7	66.6
5	11.9	36.7	7.7	69.6
6	17.9	42.4	11.7	61.1
7	14.7	82.2	9.7	96.5
8	17.5	87.1	14.1	139.6

SS: Steady State.

**Table 3:** **Estimated time constants and normalized steady state gains of VO**_**2**_** response**

**Subject**	**Onset exercise**		**Offset exercise**	
	**SS Gain (k**_**1**_**)**	**Time constant (*τ***_**1**_**)(sec)**	**SS Gain (k**_**2**_**)**	**Time constant (*τ***_**2**_**)(sec)**
1	303.2	40.5	261.8	40.5
2	319.4	40.3	313.6	64.8
3	345.3	56.2	340.6	71.4
4	409.3	44.4	383.9	61.6
5	286.5	30.4	283.0	56.5
6	273.9	39.7	247.0	44.1
7	368.5	63.4	367.1	71.7
8	304.7	66.0	291.6	98.4

SS: Steady State

By observing the original data for each subject we can clearly identify that all subjects have similar HR and VO_2_ profiles. For each individual subject, steady state gain of offset is smaller than steady state gain of onset for both HR and VO_2_.

Average values of onset and offset time constants as well as steady state gains for VO_2_ and HR profiles are shown in Table 4. These values will be used to simulate the VO_2_ and HR responses during the proposed interval training protocol.

**Table 4:** Averaged values of time constants and steady state gains

**Profile**	**Onset**		**Offset**	
	***τ***_**1**_	**k**_**1**_	***τ***_**2**_	**k**_**2**_
HR	64.9	15.4	94.9	11.3
**SD**	18.8	2.0	26.6	2.2
VO_2_	47.6	326.4	63.6	311
**SD**	11.9	42.5	17.1	46.1

SD: Standard Deviation

Based on the results listed in Tables 2, 3 and 4, it can be seen that the time constant of offset is larger than that of onset for both HR and VO_2_. Paper [16] has shown the same result for HR response during walking exercises; nevertheless, it did not investigate the dynamic characteristics of a running protocol.

### The proposed interval training protocol

In this subsection, a sensible interval training protocol will be built based on the experimental results. To build a training protocol, targets have to be set first.

For instance, if the aim of a training protocol is to develop basic endurance and aerobic capacity, all running intervals should be completed at a maximum of 70% of HR_max_ while developing cardiovascular system can be done by training at a range of 70% to 80% of HR_max_.

On the other hand, training in anaerobic zone which is 80% to 90% of HR_max_ will develop the lactic acid system [23]. Therefore, determining an actual HR_max_ is the key to constructing a well-designed training program. According to [24], a population specific formula should be used for better estimation of HR_max_ for an individual subject. However, the most accurate general equation to estimate HR_max_ is that of Inbar [25]:

(2) $${\text{HR}}_{\text{max}}=205.8-0.685\times (\text{Age})$$

It is well known that HR is a valid measure of exercise intensity only if it reflects the metabolic rate which can be measured by VO_2_. Improving aerobic capacity can be achieved by increasing oxygen delivered to the muscles during exercise. Table 5 shows the relationship between VO_2max_ and HR_max_[3].

**Table 5:** **Relationship between HR**_**max**_** and VO**_**2max**_

**%HR**_**max**_	**%VO**_**2max**_
<60	<40
60-70	41-55
71-80	56-70
>80	>70

Table 6 shows the training zones and their corresponding value for both HR_max_ and VO_2max_.

**Table 6:** **Exercise intensity levels that coincide with HR**_**max**_** and VO**_**2max**_

**Category**	**%HR**_**max**_	**%VO**_**2max**_
Fat burning (low)	<70	<50
Aerobic (moderate)	70-80	∼50-70
Anaerobic (very vigorous)	>80	>70

As mentioned before, a linear relationship between VO_2_ and HR exists during moderate intense activity and that is why our training protocol will target the aerobic zone (70-80% of HR_max_ or 50-70% VO_2_max) which aims to develop the exerciser’s cardiovascular system. Based on equation (2), for an averaged model HR_max_ = 205.8-0.685 × (average age) = 205.8-0.685 × (30.63) = 184.82 ≈ 185 bpm. Thus, building an interval training which reaches a 148 bpm (80% of HR_max_) in each interval will guarantee achieving ∼70% of VO_2max_.

The experiment results show that for an average model, the exerciser exceeds an 80% of his HR_max_ at the 297^th^ second which means 57 seconds after the start of the running period, also 1 minute at speed 5km/h is considered to be the recovery period after each running interval.

**Figure 3:**
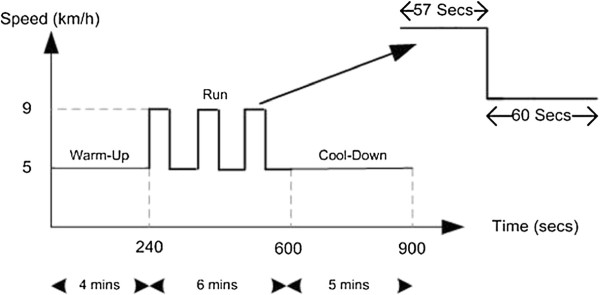
Proposed interval training protocol targeting an aerobic zone for an averaged model.

Figure 3 shows an interval training protocol based on the above calculations.

In order to verify the new proposed interval training protocol shown in Figure 3, more subjects need to be recruited, and this will be the next step of this study.

**Figure 4:**
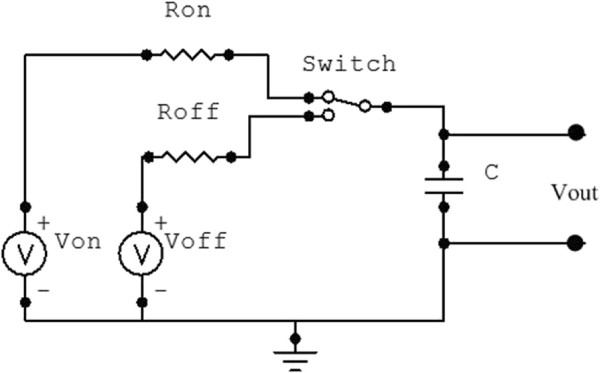
**Designed RC circuit that can be used in simulating HR and VO**_**2**_** responses to interval training protocol.**

Before verifying this protocol by experiment, we tested it by simulation first. Switching RC circuit as shown in Figure 4 was used to simulate both HR and VO_2_ responses.

The parameters of the model are tuned based on Table 4. R_On_ multiplied by C (R_On_ ×C) represents the time constant for the onset response, while R_Off_ multiplied by C (R_Off_ ×C) represents the time constant for the offset response.

The gain value can be controlled by manipulation of the voltage sources V_On_ and V_Off_. It should be noted that when the steady state gains of the onset and offset dynamics are approximately the same, V_On_ will be constant and V_Off_ is constant zero. As another special case, if both the onset and offset exercises reach steady state, then both V_On_ and V_Off_ can be constant.

The switch is utilized to replicate the switching behavior between the two responses based on the training protocol segments. National Instruments Multisim has been used to simulate the proposed interval training protocol.

**Figure 5:**
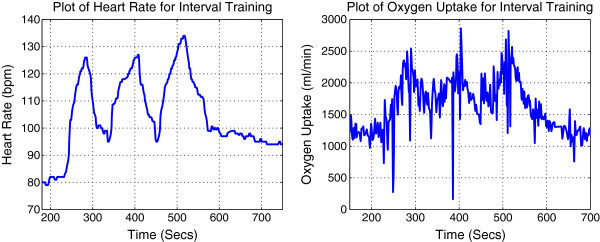
**HR and VO**_**2**_** experimental results for subject No.9 under the proposed interval training protocol.**

New male subject (Subject No.9, Age = 30 year, Weight = 84 Kg, and Height = 185 cm) was asked to run on the treadmill under the new interval training protocol. Experimental results for subject 9 are shown in Figure 5.

The experimental results approximately match the simulation results. However, in contrast with HR, the experimental results for VO_2_ were skewed due to the presence of extensive noise. As the intensity of this protocol is anticipated in the moderate range for the purpose of improving cardiovascular fitness, the relationship between HR and VO_2_ is nearly linear [2]. Furthermore, HR is easier to be measured and has wider frequency bandwidth. Therefore, HR is suitable and therefore recommended to be used as an indicator of exercise strength for the proposed interval training protocol.

## Individualized adaptation for the proposed interval training protocol

The proposed interval training protocol is based on the established average model for eight healthy young male subjects. However, for an individual exerciser (who is young and healthy) the proposed protocol may need to be adjusted due to the differences of the intra- and inter-subjects.

In this section, we present a hybrid system model (a dynamic system that exhibits both continuous and discrete dynamic behaviors) to describe the adaptation process and propose a multi-loop PI (Proportional and Integral) control approach for the tuning of interval training protocol.

### The adaptation framework

**Figure 6:**
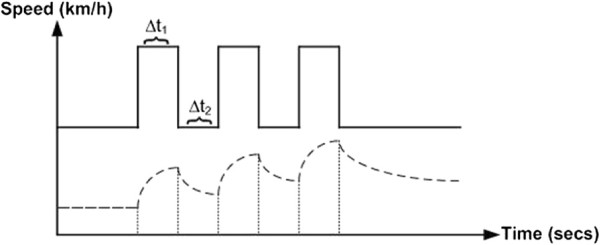
**Controller outputs’ inputs (duty cycle (*Δ* t**_**1**_**) and period(*Δ* t**_**1**_** +** ***Δ* t**_**2**_**)) and HR response during interval training protocol.**

The final goal of the adaptation of the proposed interval training protocol is tuning the duty cycle (*Δ* t_1_) as shown in Figure 6, and the period (*Δ* t_1_ + *Δ* t_2_) to achieve desired training effects. The adaptation process is a set of successive interval training experiments for a particular user. This process can be described by using a hybrid system model [26].

Specifically, each single experiment can be considered as a discrete event and the dynamics of cardio-respiratory responses to exercise can be depicted by a continuous linear model. It will be shown under modest assumptions, we can simplify this special hybrid system as a simple discrete time system.

The intermission between the interval training experiments can be hours or days. However, in order to simplify the process, we first assume that the intermission is constant (similar as the sampling time of a discrete time system) given that the subject has similar physiological conditions before each training (i.e., the intermission between the interval training is long enough to recover the exercisers).

For each training, the cardio respiratory responses (HR/VO_2_) are actually the outputs of continuous systems. In order to simplify the system as a static discrete time system, we first pick up some key characteristics from the continuous processes, which are associated with exercise effects, such as mean value and standard deviation. The proposed training protocol (see Figure 3) contains three-period square wave. In this study we pick up the lowest point and the highest point of the third period HR response y(t_4_) and y(t_5_) respectively as the reflections of exercise effects (see Figure 6).

We can then treat every single interval training experiment as a simple static system, which has two inputs (the duty cycle (*Δ* t_1_) and the period (*Δ* t_1_ + *Δ* t_2_)) and two outputs (y(t_4_) and y(t_5_)). Under the above assumptions and simplification, the overall adaptation process can now be simply treated as a two-input two-output (2I2O) discrete time system.

In order to determine the model of the 2I2O static system, we investigate HR response for the proposed interval training protocol. Figure 6 shows the protocol and its response.

Based on the previous model, the outputs y(t_4_) and y(t_5_) can be obtained as follows:

(3) $$\left\{\begin{array}{l} y_{\text{HR}}(t_{4})=y_{\text{HR}}(t_{3}).(1-(k_{2}/k_{1})(1-\;e^{\frac{-\Delta t_{2}}{T_{2}}}))\\ y_{\text{HR}}(t_{5})=y_{\text{HR}}(t_{1})+y_{\text{HR}}(t_{4}).\;e^{\frac{-\Delta t_{1}}{T_{1}}} \end{array}\right)$$

Where k_1_, k_2_, T_1_ and T_2_ are the onset, offset gains and time constants respectively, and

(4) $$\left\{\begin{array}{l} y_{\text{HR}}(t_{1})=k_{1}.(1-\;e^{\frac{-\Delta t_{1}}{T_{1}}})\\ y_{\text{HR}}(t_{2})=y_{\text{HR}}(t_{1})-k_{2}.(1-\;e^{\frac{-\Delta t_{1}}{T_{1}}}).(1-\;e^{\frac{-\Delta t_{2}}{T_{2}}})\\ y_{\text{HR}}(t_{3})=y_{\text{HR}}(t_{1})+y_{\text{HR}}(t_{2}).\;e^{\frac{-\Delta t_{1}}{T_{1}}} \end{array}\right)$$

### Controller design and simulation study

As the adaptation can be depicted as a discrete time system, we can design a discrete time multi-loop controller (Proportional and Integral controller) to adjust the two inputs (the duty cycle (*Δ* t_1_) and the period (*Δ* t_1_ + *Δ* t_2_)) to regulate the two outputs (y(t_4_) and y(t_5_)) to reach the desired reference values.

**Figure 7:**
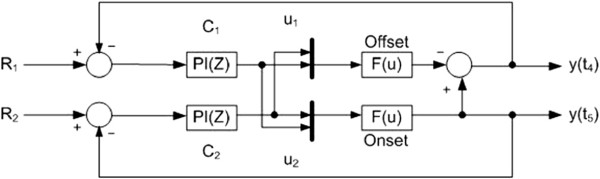
**Multi-loop PI control system to regulate output values y(t**_**4**_**) and y(t**_**5**_**).**

Figure 7 is the block diagram of the proposed control system. Two discrete PI controllers C_1_ and C_2_ have been built and connected to the HR model to control the desired output values y(t_4_) and y(t_5_) separately. The controller parameters (the coefficients of P and I actions) of these two simple multi PI controllers are tuned by trial and error. We simulated the control performance in Matlab Simulink.

Up to now, we can see even though our aim is to regulate a continuous process; we proposed a simple discrete time control technique to fulfill the goal.

During interval training of an individual subject, at the first iteration the exerciser is asked to run under the predefined training protocol. HR response at t_4_ and t_5_ (as shown in Figure 6) will then be measured and feedbacked to the multi-loop control system to update controller outputs’ inputs u_1_ and u_2_ (the duty cycle (*Δ* t_1_) and the period (*Δ* t_1_ + *Δ* t_2_)).

This modification will try to adjust the measured output values (y(t_4_) and y(t_5_)) to approach the desired setpoints for the next training exercise. This recursive process will be repeated until we finally reach the desired set points.

**Figure 8:**
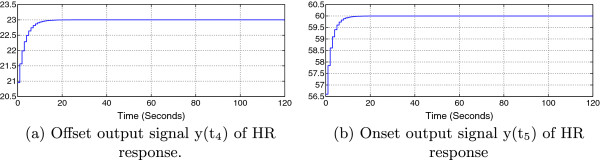
**Simulation results of PI control system outputs.** Offset output signal y(t_4_) of HR response **(a)** and onset output signal y(t_5_) of HR response **(b)**.

The self-adaption feature gives the exerciser the opportunity to reach his desired setpoints after a number of iterations. Figure 8 below shows the simulated controller outputs, y(t_4_) and y(t_5_).

In simulation, from Figure 8, we conclude that after at least 12 iterations (12 training exercises) the system will reach the desired setpoints.

**Figure 9:**
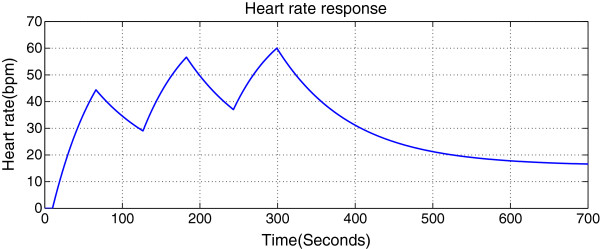
Continuous interval training HR response simulation from the controller.

In other words, t_4_ and t_5_ have to be observed 12 times and re-entered to the controller to achieve the desired outputs. Figure 9 below shows the HR response for the 13^th^ iteration. The third waveform in Figure 9 shows that the output signal finally reached the desired reference values R_1_ and R_2_ at t_4_ and t_5_ respectively.

Since our controller is based on discrete events regulating a continuous process, it can perform one time between two training experiments. Therefore, it is very easy to be implemented in low cost portable devices which have limited computation power.

## Control system verification

This section provides a practical method to examine and verify the functionality as well as performance of the proposed controller. The new interval training protocol combined with the designed controller have been implemented and tested on stair climbing exercise as a free environment activity. This can show how the proposed interval training protocol that has been created earlier can be implemented using different exercising techniques, such as stairs climbing, cycling and even swimming.

The setup, concept and structure of the interval training remain the same. However, the change would be within the type of exercise during the onset and offset stages. Subjects in the new implementation will be requested to ascend a staircase of steps without any pause; the exercisers will attempt to achieve the training protocol without holding the banisters or the handrail.

The experiments were carried out at free speed and the participants were given the instruction to walk at their normal pace. However, each exerciser was instructed to stay within 4-5 km/h climbing speed. This will represent the onset stage in our training protocol. On the other hand, offset stages can be represented as walking aside on the same step the exercisers reach when the onset period finishes; this continuous walking on the same step is the perfect match of the offset stage in the treadmill interval training exercise. All participants were instructed to use their left legs for the first step, to only place one foot on each step (foot-over-foot ascent) and to continue walking in straight line.

Based on the simulation results in the previous section, the exerciser needs 12 iterations to reach his desired setpoints. Practically, the experiments results for a new male subject (Subject No.10, Age=31 year) show that the participant has almost reached his desired setpoints after three iterations only.

Based on the exerciser’s age, the controller R_1_ and R_2_ are set to be 148 bpm(80% of HR_max_) and 111 bpm(60% of HR_max_) respectively. In the first iteration, 60 seconds is set to be the onset and offset times. At the end of the first training session, the reading of HR at t_4_ and t_5_ were observed and feed backed to the multi-loop control system to update the controller output’s inputs (the duty cycle and the period). Accordingly, the controller adjusts the time of onset and offset periods for the next training session. Table 7 shows the controller parameters and their corresponding experimental results for Subject No.10 in three training sessions.

**Table 7:** Control system parameters and their corresponding experimental results for subject no.10 after three iterations

**Iteration**	**Onset (sec)**	**Offset (sec)**	**Period (sec)**	**Duty cycle (%)**	**y(t**_**4**_**)(bpm)**	**y(t**_**5**_**)(bpm)**
Ref.	60	60	120	50	111	148
1	53	55.8	108.8	48.7	115	136
2	54.1	56.2	110.3	49.1	116	143
3	54.6	56.4	110.9	49.2	118	148

**Figure 10:**
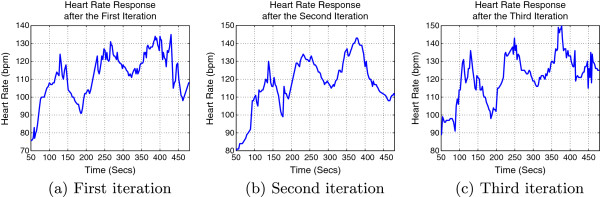
**Subject 10’s HR responses under stairs climbing exercise.** HR response after the first iteration **(a)**, HR response after the second iteration **(b)** and HR response after the third iteration **(c)**.

Figure 10 shows the new subject’s HR response under the stairs climbing activity.

Referring to the results from Table 7 and Figure 10, we can conclude that the exerciser has almost reached his desired setpoints after only 3 iterations, when the simulation results showed that it can be done after 12 iterations. This shows how our controller has accomplished its duty with minimum number of iterations.

It is worth mentioning that for a proposed training regime based on an averaged model; we expect each subject to respond differently to a particular exercise or training protocol, of interest was Subject 10 who reached his desired set points after only three iterations. We may reach the desired set points or a steady state for another subject after 10, 11 or even 12 iterations, it depends totally on the intra- and inter- model uncertainties.

During the study, it was considered that after a certain number of training sessions, the training capacity of the subject will improve and this may affect the result of his HR response. In the next step of the study, we will recruit more subjects in order to verify our results and to even improve the performance of our controller.

## Conclusion

A designed square-wave exercise protocol was applied to investigate the dynamic characteristics of HR and VO_2_ responses for both the onset and offset exercises. The K_4_b^2^ portable device was used to measure breath-by-breath VO_2_ and beat-by-beat HR. According to the experimental results, it was concluded that the time constants and steady state gains of the onset exercise for both HR and VO_2_ are distinctively different with those of the offset exercise. Based on the identified dynamic characteristics for both HR and VO_2_, we proposed an interval training protocol to improve cardiovascular fitness. A switching RC circuit model was presented to simulate HR and VO_2_ responses for the proposed interval training protocol. In order to adapt to individual users, a multi-loop integral control scheme has been proposed to regulate the HR response for interval running exercise under free living conditions. In the next step of the study, we will recruit more subjects for the validation of the proposed models, interval training protocol and the feedback control based adaption scheme.

## Competing interests

The authors declare that they have no competing interests.

## Authors’ contributions

AH carried out the experiments and drafted the paper; YZ carried out the simulation part. SS supervised the overall project and the revision of the paper. BC supervised the project. HN supervised the project and revised the paper. All authors read and approved the final manuscript.
